# Performance differences among commercially available antigen rapid tests for COVID-19 in Brazil

**DOI:** 10.1371/journal.pone.0269997

**Published:** 2022-06-16

**Authors:** Mariana Lourenço Freire, Lindicy Leidicy Alves, Carolina Senra de Souza, Juliana Wilke Saliba, Verônica Faria, Mariana Junqueira Pedras, Nara de Oliveira Carvalho, Gláucia Queiroz Andrade, Ana Rabello, Daniel Moreira Avelar, Gláucia Cota

**Affiliations:** 1 Pesquisa Clínica e Políticas Públicas em Doenças Infecciosas e Parasitárias, Instituto René Rachou, Fundação Oswaldo Cruz, Belo Horizonte, Minas Gerais, Brazil; 2 Coordenação Estadual de Laboratórios e Pesquisa em Vigilância da Subsecretaria de Vigilância em Saúde da Secretaria do Estado da Saúde de Minas Gerais; 3 Núcleo de Ações e Pesquisa em apoio diagnóstico (NUPAD), Faculdade de Medicina, Universidade Federal de Minas Gerais (UFMG), Belo Horizonte, Minas Gerais, Brazil; Consejo Nacional de Investigaciones Cientificas y Tecnicas, ARGENTINA

## Abstract

A rapid and accurate diagnosis is a crucial strategy for containing the coronavirus disease (COVID-19) pandemic. Considering the obstacles to upscaling the use of RT–qPCR, rapid tests based on antigen detection (Ag-RDT) have become an alternative to enhance mass testing, reducing the time for a prompt diagnosis and virus spreading. However, the performances of several commercially available Ag-RDTs have not yet been evaluated in several countries. Here, we evaluate the performance of eight Ag-RDTs available in Brazil to diagnose COVID-19. Patients admitted to tertiary hospitals with moderate or mild COVID-19 symptoms and presenting risk factors for severe disease were included. The tests were performed using a masked protocol, strictly following the manufacturer’s recommendations and were compared with RT–qPCR. The overall sensitivity of the tests ranged from 9.8 to 81.1%, and specificity greater than 83% was observed for all the evaluated tests. Overall, slight or fair agreement was observed between Ag-RDTs and RT–PCR, except for the Ag-RDT COVID-19 (Acro Biotech), in which moderate agreement was observed. Lower sensitivity of Ag-RDTs was observed for patients with cycle threshold > 25, indicating that the sensitivity was directly affected by viral load, whereas the effect of the disease duration was unclear. Despite the lower sensitivity of Ag-RDTs compared with RT–qPCR, its easy fulfillment and promptness still justify its use, even at hospital admission. However, the main advantage of Ag-RDTs seems to be the possibility of increasing access to the diagnosis of COVID-19 in patients with a high viral load, allowing immediate clinical management and reduction of infectivity and community transmission.

## Introduction

Initially described in China in December 2019, coronavirus disease (COVID-19), caused by severe acute respiratory syndrome coronavirus 2 (SARS-CoV-2), rapidly spread worldwide, becoming a major global health concern, and causing massive socioeconomic disruption [1, 2]. Although the rapid development and availability of vaccines demonstrate the enormous technical-scientific response capacity, operationally, worldwide vaccination faces serious challenges to overcome the economic inequalities between countries and the crescent, but not recent, phenomenon of vaccine rejection. Furthermore, the mutagenic capacity of the virus and continuous emergence of variants make full control of the pandemic a goal not yet achieved. Accurate diagnosis tests for SARS-CoV-2 remain necessary for monitoring and containing new waves of COVID-19 by the early diagnosis of cases, minimizing opportunities for transmission.

Real-time reverse transcription polymerase chain reaction (RT–qPCR) using respiratory specimens has been recommended as a reference diagnostic test for acute SARS-CoV-2 infection and has been widely implemented [3]. Considering tests with high sensitivity and specificity, they are challenging to implement at scale, particularly in resource-poor settings: they are costly, time-spending and require laboratory infrastructure and highly trained technicians [4, 5].

Antigen-based tests (Ag-RDTs) quickly emerged as a viable alternative to large-scale testing for SARS-CoV-2. Although they are inferior to RT–qPCR in terms of sensitivity and specificity [6, 7], their potential advantages of ease of execution, low cost and short time until results, at the point of care without the need for a laboratory, make them tools of choice in favor of a timely decision-making process [8]. However, for Ag-RDT to fulfill this purpose, it is essential to recognize the performance of different tests and the impact of disease duration and other variables on their accuracy in real-life studies in different world regions. Significant differences between the accuracy of Ag-RDT reported by the manufacturers and that observed under field conditions have already been reported [9, 10]. Most of these studies evaluated hospitalized patients with severe forms of COVID-19 and in the first days of symptoms, conditions with a presumed high viral load in respiratory specimens, which possibly favors test performance [11, 12]. Herein, we aimed to evaluate in parallel the performance of eight Ag-RDTs commercially available in Brazil in patients presenting mild respiratory symptoms and COVID-19 suspicion.

## Methods

This study was performed according to the Standards for Reporting of Diagnostic Accuracy (STARD) statement [13].

### Study design, sample processing and diagnostic procedures

This prospective study was conducted in a tertiary hospital setting in the municipality of Belo Horizonte, state of Minas Gerais, Brazil. Patients presenting a clinical suspicion of COVID-19 were consecutively enrolled and tested on hospital admission by RT–qPCR for SARS-CoV-2. Only patients with a mild clinical condition who were eligible for noninvasive treatment were included in this study. For each recruited participant, two samples were collected through consecutive paired nasopharyngeal swabs, one from each nostril. Depending on the manufacturers’ requirements, the rapid test was performed at the bedside immediately after collection (maximum of 30 minutes) or, if explicitly authorized, could be performed using the Universal Transport Medium (Copan UTM system; Copan, Italy; catalog no. 3C047N) in the reference laboratory at a maximum of 12 hours after sampling. Thus, using the UTM, from the clinical specimen obtained from a single participant it was possible to perform several tests, limited by the total volume of the transport solution available. The UTM volume was always limited to a maximum of 2 mL to minimize sample dilution. Therefore, whenever authorized by the manufacturer, the UTM was the preferred source of performing the test. Thus, considering these two tests execution possibilities, we developed a study schedule based on two phases. In the first phase, the swab used in the first nostril was kept in transport medium and, from this solution the RT–qPCR and four Ag-RDTs were performed (randomly chosen among the six Ag-RDTs UTM-enabled). The swab used in the second nostril was directly used in the execution of one Ag-RDT (randomly chosen among the two Ag-RDTs based on nasopharyngeal samples whose manufacturers did not allow execution with UTM). As the same way, in the second phase, the swab used in the first nostril was immersed in UTM, which was used in the execution of the RT–qPCR and the other two Ag-RDTs UTM-enabled. The swab used in the second nostril was directly used in the execution of one Ag-RDT, and the patients were submitted to a new collection, this time covering only the nasal mucosa for execution of a commercial test based on exclusive nasal swab collection (and not nasopharyngeal). The number of participants varied depending on the total patients recruited at each study phase, which ended when the minimum number of samples required (52 patients, according to the sample calculation) was reached at the end of the day. At the end of each study phase, depending on the availability of Ag-RDTs, two additional days of recruitment and testing strategy were maintained in order to gather some patients to be tested simultaneously using dry swab and UTM sources, a secondary analysis. During all the study, RT–qPCR results were masked to all researchers involved in the rapid test reading.

### Ethics approval

Ethical approval was obtained from Instituto René Rachou, Fundação Oswaldo Cruz, CAAE: 30960120.0.0000.5091 (Number 4.001.133) and Eduardo de Menezes Hospital, Fundação Hospitalar do Estado de Minas Gerais, CAAE 42314921.0.3001.5124 (Number 4.595.768). The samples were used only after written formal acceptance of the participants over than 21 years old.

### Sample calculation

For a comparison based on agreement with the reference test (RT–qPCR), the minimum population required for one test validation was estimated as 52 patients, according to Arifin et al. (2021) [14]. The premises were a power of 80%, a significance level of 5%, a minimum disease prevalence in the sample of 50% and a minimum acceptable Kappa of 0.5, with an expected Kappa of 0.8. Patients with undetermined RT–qPCR results were excluded from this analysis.

### Selection and execution of antigen-detection rapid diagnostic tests (Ag-RDTs)

All manufacturers of the 58 Ag-RDTs for COVID-19 registered at the Brazilian National Health Surveillance Agency (ANVISA) until March 2021 were contacted and invited to participate in this validation study. Of the 16 tests whose manufacturers agreed to participate in this validation, eight were selected based on the availability of supplying the kits within 40 days and possibility of execution from transport medium using a minimum required volume. All the tests were performed strictly according to the manufacturer’s instructions using the buffer provided in each kit or UTM. The main characteristics of the selected Ag-RDTs are shown in Table 1 and include information concerning UTM use.

**Table 1:** Antigen-detection rapid diagnostic tests (Ag-RDTs) and their main characteristics, including the use of universal transport medium (UTM).

Characteristic	COVID-19 Ag ECO Teste	SARS-CoV-2 Ag-RDT	CORIS Bioconcept^®^ COVID-19 Ag-RDT	CELLER WONDFO SARSCOV2 Ag-RDT	NowCheck COVID-19 Ag test	Ag-RDT COVID-19	Panbio^™^ COVID-19 Ag-RDT	Panbio^™^ COVID-19 Ag-RDT Device
Manufacturer	Eco Diagnostica	SD Biosensor	Nanosens	Guangzhou	Bionote	Acro Biotech	Abbott Rapid	Abbott Rapid
Country	Brazil	South Korea	Belgium	China	South Korea	United States	Germany	Germany
Antigen detected	Not specified	Not specified	N protein	Not specified	Not specified	Not specified	Not specified	N protein
Specimen	nasopharyngeal swab	nasopharyngeal swab	nasopharyngeal swab	nasopharyngeal swab	nasopharyngeal swab	nasopharyngeal swab	nasopharyngeal swab	nasal swab
Use of UTM*/volume	Yes/350 μL	Yes/350 μL	Yes/100 μL	Yes/80 μL	Yes/350 μL	NA	NA	NA
Time to result	15–30 minutes	15–30 minutes	30 minutes	15–20 minutes	15–30 minutes	15–20 minutes	15–20 minutes	15–20 minutes

* Universal Transport Medium COPAN Diagnostics Inc.; NA: not applicable.

For tests performed immediately after sampling, the nasopharyngeal swab was immersed in the buffer solution and then dripped onto the test plate. For tests performed using samples stored in UTM, this medium was diluted in the buffer, and the final solution was dripped in the appropriate place on the reagent strip. In both cases, after the recommended waiting time, the control and test bands were observed in the test membrane. The test was considered positive if the control band was reactive and any intensity band was observed at the test band.

### RT–qPCR

RNA extraction was performed using a MagMax Viral/Pathogen Nucleic Acid Isolation Kit (ThermoFisher Scientific, Waltham, MA, USA) or a Chemagic Viral DNA/RNA H96 kit (PerkinElmer, Waltham, MA, USA), and amplification was performed using a commercial rRT-qPCR kit (TaqPath COVID-19 CE-IVD RT–qPCR; ThermoFisher Scientific) containing ORF1ab, Nucleocapsid (N) and Spike (S) as target sequences for SARS-CoV-2. All RT-qPCRs were performed using QuantStudio 5 (ThermoFisher Scientific, Waltham, MA, USA). A cycle threshold (CT) value of 37 was designated the cutoff value for positive results. Amplifications in at least two target regions of SARS-CoV-2 were considered positive, and the absence of an amplification signal was considered negative. Any other RT–qPCR results were considered inconclusive.

### Data analysis

Descriptive statistics were used to present the main characteristics of the population. We used the Shapiro–Wilk normality test to evaluate whether the data were normally distributed. Continuous variables were presented as medians and interquartile range (IQR 25–75%), and the Mann–Whitney U test was used to compare medians. Categorical variables, expressed as numbers (percentages), were compared by chi-squared test or Fischer’s exact test as appropriate. The accuracy analyses of Ag-RDT tests were determined according to RT-qPCR result using MedCalc Software (Version 20.015). Based on a two-by-two contingency table, sensitivity was considered as the number of true positive patients on the Ag-RDTs divided by the total of positive patients on the RT-qPCR, and specificity was considered the number of true negative patients on the Ag-RDTs divided by the total of negative patients on the RT-qPCR. Finally, accuracy was determined by the number of RT–qPCR and Ag-RDT concordant results divided by the total number of tested patients. RT–qPCR was defined as the reference standard, and the agreement was calculated using the Kappa index and interpreted following the criteria of Landis and Koch (1977) [15] as follows: <0, no agreement; 0–0.2, slight agreement; 0.2–0.4, fair agreement, 0.4–0.6, moderate agreement; 0.6–0.8, substantial agreement; 0.8–1, almost perfect agreement. The sensitivity, specificity, accuracy and Kappa index were presented with 95% confidence intervals (95% CIs). For each Ag-RDT, complementary analyses were performed stratifying patients into days of symptoms (7 days) and the total CT mean using logistic regression with Minitab Statistical Software. Although the Ct mean gathering the three genes’ targets has no diagnostic meaning, we have used this value as proxy for the magnitude of the total viral load in the sample, a mathematical strategy to correlate the viral load with test performances.

## Results

### Population characteristics

A total of 162 nonvaccinated participants were included from March 22 to April 21, 2021. The flow diagram showing the patients included in the study and its primary outcome according to the test is presented in the S1 Fig. The median age of the participants was 56.3 years (IQ 25–75%: 46–65 years), and 53.7% were female. The median duration of symptoms was 9.8 days (IQ 25–75%: 6–13 days), and the most frequent clinical manifestations were cough (84.6%), shortness of breath (74.1%) and fever (67.3%), followed by myalgia (62.4%), headache (54.9%) and diarrhea (43.2%). The most commonly observed comorbidity was hypertension, which was present in 51.2% of the patients, followed by diabetes (29.6%) and respiratory chronic diseases (9.9%). Forty-nine hospitalized patients (30.2%) did not present any comorbidities. The prevalence of SARS-CoV-2 infection defined by RT–qPCR positivity was 80.9%, comprising 131 confirmed patients, with a mean Ct of 23.5 (interquartile range [IQR]: 19.7–27.4]. The characteristics of each population according to Ag-RDT are detailed in Table 2.

**Table 2:** Population characteristics of each evaluated test.

Characteristic	COVID-19 Ag ECO Teste (Eco Diagnostica)	SARS-CoV-2 Ag-RDT (SD Biosensor)	CORIS Bioconcept^®^ COVID-19 Ag-RDT (Nanosens)	CELLER WONDFO SARSCOV2 Ag-RDT (Guangzhou)	NowCheck COVID-19 Ag test (Bionote)	Ag-RDT COVID-19 (Acro Biotech)	Panbio^™^ COVID-19 Ag-RDT- Nasopharyngeal (Abbott Rapid)	Panbio^™^ COVID-19 Ag-RDT Device—Nasal (Abbott Rapid)
**n**	**81**	**81**	**76**	**63**	**64**	**66**	**65**	**65**
**Median age [IQR]**	**56.2 [47–64.5]**	**57.3 [47–66]**	**56.3 [47–65.5]**	**55.4 [44–64]**	**55.6 [44–64]**	**56.2 [47–64]**	**56.1 [44–64,3]**	**56.1 [44–64,3]**
**Sex**								
Female (%)	49 (60.5%)	48 (59.3%)	46 (60.5%)	29 (46.1%)	31(48.4%)	39 (59.1%)	31 (47.7%)	31 (47.7%)
Male (%)	34 (39.5%)	33 (40.7%)	30 (39.5%)	34 (53.9%)	33 (51.6%)	27 (40.9%)	34 (52.3%)	34 (52.3%)
**Symptom**								
Fever	47	48	47	51	51	40	51	51
Cough	71	72	69	49	50	59	51	51
Sore throat	32	30	27	23	23	24	23	23
Coryza	28	28	24	21	21	23	21	21
Headache	42	43	42	35	36	35	36	36
Body ache	50	52	53	39	39	45	39	39
Diarrhea	30	30	29	31	31	24	31	31
Loss of small	31	31	31	22	22	25	22	22
Loss of taste	33	34	32	25	25	25	25	25
Shortness of Breath	64	63	60	44	44	51	45	45
**Symptom duration (days)**								
1–3	9	9	9	9	9	8	9	9
4–7	24	26	20	17	18	17	18	18
8–13	26	26	25	24	25	23	25	25
>14	22	20	22	13	12	18	13	13
**mean [IQR]**	**9.4 [6–14]**	**9.0 [5–13,3]**	**10.2 [6–14]**	**9.8 [6.5–12]**	**9.7 [6–12]**	**9.8 [6–13]**	**9.8 [6–13]**	**9.8 [6–13]**
**Ct value** *								
Orf1ab (mean [IQR])	24.3 [20.8–28.1]	24.3 [20.8–28.2]	24.3 [20.8–28.2]	21.9 [17.7–24.9]	22.0 [18.0–25.0]	24.1 [20.7–28]	22 [18–25]	22 [18–25]
S (mean [IQR])	25.2 [21.1–28.9]	25.3 [21.1–29.2]	25.3 [21.1–29.2]	22.5 [18.6–25.5]	22.6 [18.8–25.5]	25 [20.9–28.8]	22.6 [18.8–25.6]	22.6 [18.8–25.6]
N (mean [IQR])	24.7 [21.2–27.8]	24.7 [21.2–28.0]	24.7 [21.2–28.0]	22.4 [19.1–25.7]	22.5 [19.2–25.8]	24.5 [21.2–27.8]	22.5 [19.2–25.8]	22.5 [19.2–25.8]

*Based on RT–qPCR results.

### Ag-RDT results

Overall, the sensitivity of the Ag-RDTs ranged from 9.8 to 81.1%, and the specificity was higher than 83.3% for all evaluated tests (Table 3). The agreement beyond chance expressed by the Kappa index demonstrated fair agreement (K ≥0.2 < 0.4) for most Ag-RDTs except for the Ag-RDT COVID-19 (Acro Biotech) test, for which moderate agreement was observed (K = 0.53) and for CORIS Bioconcept^®^ Ag-RDT (Nanosens) that presented slight agreement (K = 0.04).

**Table 3:** Performance of antigen-detection rapid diagnostic tests for COVID-19.

Ag-RDTs		RT–qPCR			Sensitivity (95% CI)	Specificity (95% CI)	Accuracy (95% CI)	Kappa index (95% CI)
		Positive	Negative	Total				
**COVID-19 Ag ECO Teste (Eco Diagnostica)**	**Positive**	27	3	**30**	42.9% (30.5–56.0)	83.3% (58.6–96.4)	51.9% (40.5–63.1)	0.16 (0.02–0.30)
	**Negative**	36	15	**51**				
	**Total**	**63**	**18**	**81**				
**SARS-CoV-2 Ag-RDT (SD Biosensor)**	**Positive**	35	2	**37**	53.0% (40.3–65.4)	86.7% (59.5–98.3)	59.3% (47.8–70.0)	0.23 (0.08–0.38)
	**Negative**	31	13	**44**				
	**Total**	**66**	**15**	**81**				
**CORIS Bioconcept**^®^ **Ag-RDT (Nanosens)**	**Positive**	6	0	**6**	9.8% (3.7–20.2)	100% (78.2–100)	27.6% (18.0–39.0)	0.04 (0.00–0.08)
	**Negative**	55	15	**70**				
	**Total**	**61**	**15**	**76**				
**CELLER WONDFO SARSCOV2 Ag-RDT (Guangzhou)**	**Positive**	25	0	**25**	47.2% (33.3–61.4)	100% (69.2–100)	55.6% (42.5–68.1)	0.22 (0.09–0.36)
	**Negative**	28	10	**38**				
	**Total**	**53**	**10**	**63**				
**NowCheck COVID-19 Ag test (Bionote)**	**Positive**	33	0	**33**	60% (45.9–73.0)	100% (66.4–100)	65.6% (52.7–77.1)	0.30 (0.16–0.47)
	**Negative**	22	9	**31**				
	**Total**	**55**	**9**	**64**				
**Ag-RDT COVID-19 (Acro Biotech)**	**Positive**	43	2	**45**	81.1% (68.0–90.6)	84.6% (54.5–98.1)	81.8% (70.4–90.2)	0.53 (0.31–0.76)
	**Negative**	10	11	**21**				
	**Total**	**53**	**13**	**66**				
**Panbio**^**™**^ **Ag-RDT—Nasopharyngeal (Abbott Rapid)**	**Positive**	33	0	**33**	60.0% (45.9–73.0)	100% (69.2–100)	66.2% (53.4–77.4)	0.32 (0.15–0.49)
	**Negative**	22	10	**32**				
	**Total**	**55**	**10**	**65**				
**Panbio**^**™**^ **Ag-RDT Device—Nasal (Abbott Rapid)**	**Positive**	32	0	**32**	58.2% (44.1–71.4)	100% (69.2–100)	64.6% (51.8–76.1)	0.30 (0.14–0.46)
	**Negative**	23	10	**33**				
	**Total**	**55**	**10**	**65**				

The agreement between the results of three commercial tests (COVID-19 Ag ECO teste (Eco Diagnostica), Coris Bioconcept Ag-RDT (Nanosens) and SARS-CoV-2 Ag-RDT (SD BIOSENSOR)) performed on the same patients using directly obtained respiratory secretions and MTU source is shown in (S1 Table) and was considered slight or fair.

**Fig 1:**
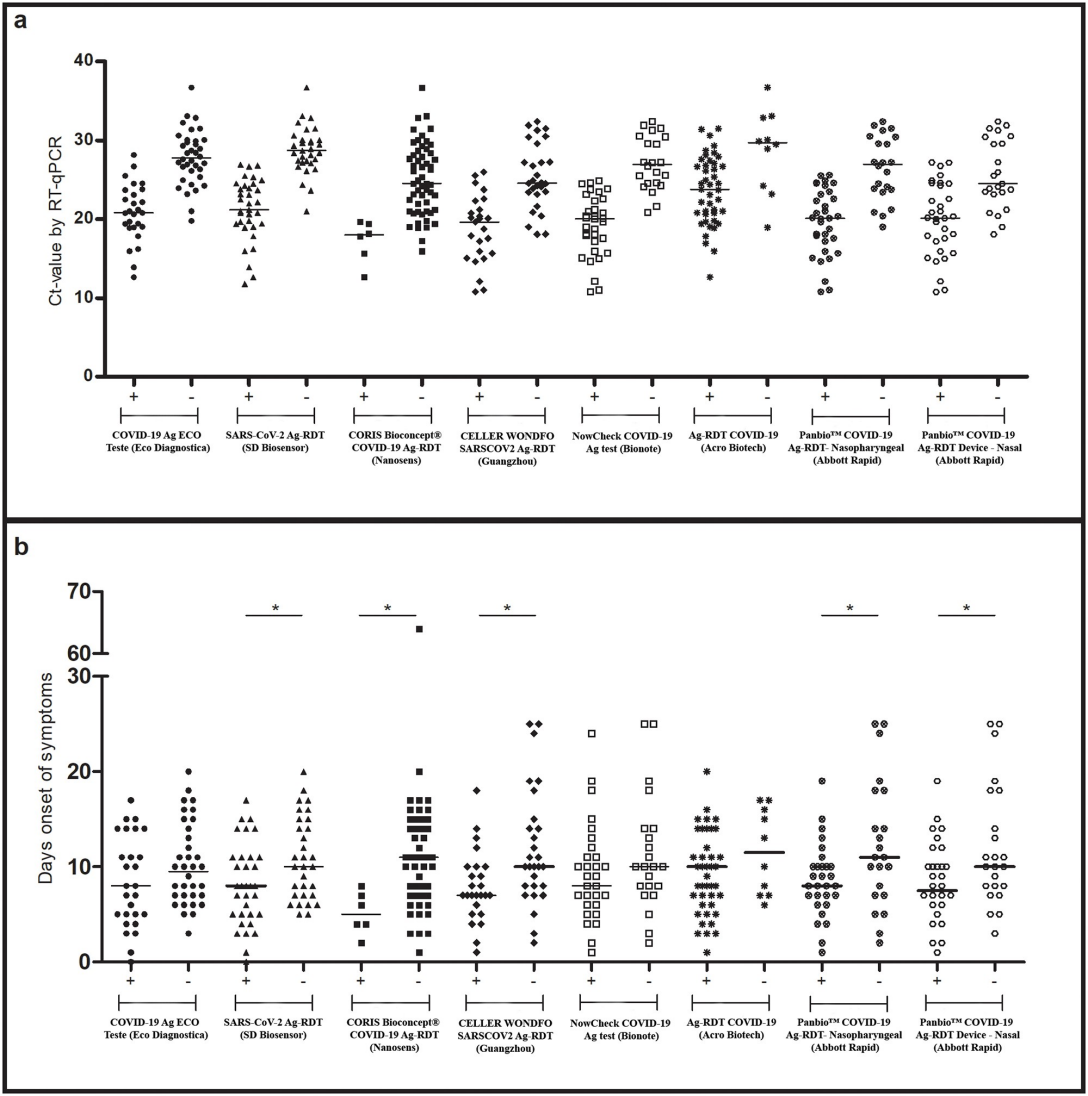
Antigen-detection rapid diagnostic test results according to the cycle threshold value observed by RT–qPCR **(a)** and days of symptom onset **(b)**. *p< 0.05; +: positive Ag-RDT; -: negative Ag-RDT.

The indirect method of quantifying viral load expressed by the Ct value, obtained by RT–qPCR, was correlated with Ag-RDT positivity. False negative Ag-RDT results were mostly observed in patients with high Ct values for all Ag-RDTs evaluated (t test; p<0.05; Fig 1a). For some Ag-RDTs, patients with more days of symptom onset had more false negative results (t test; p<0.05; Fig 1b). The greater sensitivity among patients with Ct<25 corroborates these findings (Table 4). Only for the Ag-RDT COVID-19 (Acro Biotech) test was there no statistically significant difference in sensitivity according to the Ct values. Regarding the days of symptom onset, a numerical sensitivity reduction in the second week of symptoms was observed for all Ag-RDTs, with a significant difference only for CorisBioconcept and Celler Wondfo tests.

**Table 4:** Positivity of the antigen-detection rapid diagnostic tests stratified by total Ct mean and the days of symptom onset.

	Ct average			Days of symptom onset		
	≤ 25	>25	p	≤ 7	> 7	p
**COVID-19Ag ECO teste (Eco Diagnostica)**	75.0% (24/32)	9.7% (3/31)	0.00*	54.2% (13/24)	36.9% (14/39)	0.18
**SARS-CoV-2 Ag-RDT (SD BIOSENSOR)**	90.9% (30/33)	15.2% (5/33)	0.00*	65.4% (17/26)	45.0% (18/40)	0.11
**Coris Bioconcept Ag-RDT (Nanosens)**	17.1% (6/35)	0% (0/26)	0.03*	25% (5/20)	2.4% (1/41)	0.01*
**Celler Wondfo SARSCOV2 Ag-RDT (Guangzhou)**	59.0% (23/39)	14.3% (2/14)	0.00*	70.0% (14/20)	33.3% (11/33)	0.01*
**NowCheck COVID-19 Ag test (Bionote)**	82.5% (33/40)	0% (0/15)	0.00*	76.2% (16/21)	50.0% (17/34)	0.06
**Ag-RDT COVID-19 (Acro Biotech)**	89.7% (26/29)	70.8% (17/24)	0.11	83.3% (15/18)	80.0% (28/35)	0.77
**Panbio**^**™**^ **Ag-RDT—Nasopharyngeal (Abbott Rapid)**	77.5% (31/40)	13.3% (2/15)	0.00*	71.4% (15/21)	52.9% (18/34)	0.18
**Panbio**^**™**^ **Ag-RDT Device Nasal (Abbott Rapid)**	70.0% (28/40)	26.7%(4/15)	0.01*	76.2% (16/21)	35.5% (16/34)	0.00*

*p<0.05.

**Fig 2:**
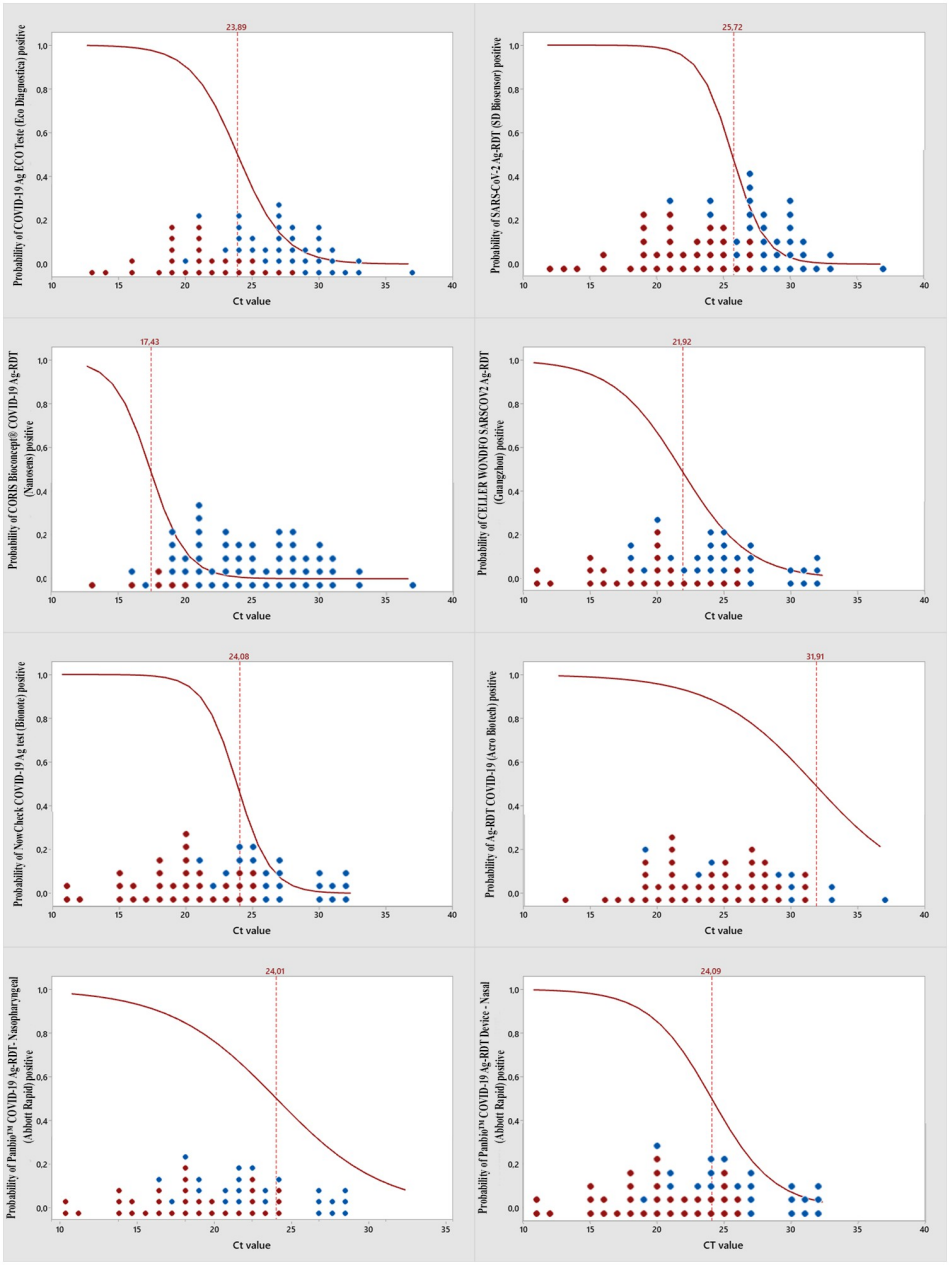
Association between the Ct value and Ag-RDT results. All dots reflect positive RT–qPCR results, shown on the x-axis at the observed mean Ct value. Red dots indicate positive Ag-RDT samples, and blue dots indicate negative Ag-RDT samples. The red line reflects the probability of a positive Ag-RDT based on the Ct value, and the red dotted line denotes the point where 50% of Ag-RDTs are expected to become positive.

Using logistic regression, the likelihood of a false negative Ag-RDT result was associated with the RT–qPCR Ct value (Fig 2). We can verify a reduction in the probability of Ag-RDT positivity with an increase in the Ct value, with 50% of Ag-RDTs expected to become positive at Ct values close to 25.

## Discussion

Because the COVID-19 pandemic remains a worldwide health problem, Ag-RDTs have arisen as important alternative diagnostic methods to RT–qPCR, increasing the possibility of mass testing, decentralizing the diagnosis and shortening the time to a confirmed diagnosis [8]. Several Ag-RDTs have been developed by various manufacturers from multiple countries and have been evaluated independently by researchers. However, most validation studies have evaluated the same tests mainly in Europe [16–18], highlighting the need for studies in countries where they are commercialized, because the population characteristics and different circulating virus variants may affect their performance [19]. Here, we evaluated the performance of eight Ag-RDTs for COVID-19 currently available in Brazil in hospitalized patients with moderate or mild disease and risk factors for severe disease requiring close monitoring.

According to the Target Product Profiles (TPP) published by WHO, Ag-RDTs should achieve ≥ 80% sensitivity and ≥ 97% specificity compared with a nucleic acid amplification test [20]. Notably, the tests evaluated here presented sensitivity ranging from 9.8 to 81.1% and specificity close to 100%, and none accomplished the recommended performance. The low sensitivity reported here may be associated with the disease stage of the patients included, in most cases in the second week of illness. However, the highest sensitivity obtained up to 7 days of symptoms was 83.3% (Table 4). The recruitment strategy in a tertiary infectious disease hospital explains the inclusion of patients in the second week of illness, a period in which clinical manifestations usually require monitoring and medical support but are characterized by decreasing viral shedding. Conversely, considering the Ct values of RT–qPCR as an indirect reference for viral load in SARS-CoV-2 infections [18], a lower sensitivity was observed for patients presenting a Ct greater than 25 in all Ag-RDTs, indicating that the sensitivity was directly affected by viral load and indirectly affected by disease length. These observations reinforce that the ideal period for the use of rapid tests may be at least until the first seven days of illness. However, the need for judicious allocation of patients in the hospital environment and diagnostic opportunity represented by attendance at the health unit when clinical symptoms intensified in the second week of illness justify the interest in evaluating the performance of antigen-based tests in this population.

The performance of Ag-RDTs may be related to the intrinsic characteristics of the patients, such as the viral load, disease severity and length of symptoms as well as the characteristics of the tests, quality of the specimen and proper handling [21, 22]. Overall, a reduced sensitivity was observed for patients presenting more than seven days of onset symptoms. However, the test with the highest sensitivity (Ag-RDT COVID-19—Acro Biotech) exhibited the same performance regardless of the number of days with symptoms, suggesting a differentiated sensitivity capable of overcoming the reduction of viral load.

Regarding sample processing, several Ag-RDT manufacturers allow the use of UTM for the temporary storage of nasopharyngeal samples. Besides the additional time gained between the sample collection and testing, the transport medium uses the same sample for the execution of different tests, which could be a strategy to allow the execution of RT–qPCR on the same specimen already collected in the case of a negative Ag-RDT test.

Even performed in accordance with the manufacturer’s instructions, one possible limitation of this strategy would be the potential of the transport medium to influence the performance of the test by promoting dilution of the clinical specimen. Here, the comparison between results obtained with the same test but performed using dry swab or UTM source revealed a worryingly weak agreement. Similar results were described by Cubas-Atienzar et al. (2021), who observed a lower sensitivity analytical limit of detection for Ag-RDT using UTM than dry swabs [23].

All evaluated Ag-RDTs are based on a sandwich immunodetection methodology with intrinsic characteristics that may affect their performance. Only two of the test manufacturers (CORIS Bioconcept Ag-RDT (Nanosens) and Panbio^™^ Ag-RDT Device Nasal (Abbott Rapid) clearly stated that the virus nucleocapsid (N) protein was used as the specific antigenic target. This structural protein is often used because of its relative abundance and because it presents the least amount of variation in the gene sequence, indicating that it is a stable protein [16, 24]. However, the presence of mutations in SARS-CoV-2 altering expression of viral proteins may potentially impact Ag-RDT performance and accuracy results in scenarios of genetic variability should be interpreted with caution. During this study, P.1 and P.2 were the variants prevalent in Brazil. Repeated validations are required in order to verify the ability of Ag-RDTs to diagnose the current circulating strains [25, 26].

Several limitations may affect the test’s accuracy. This study was conducted under the same controlled laboratory conditions (temperature and lighting). Samples were collected, tests were performed by trained staff, and a unique Ag-RDT batch was used throughout the study. The total sample of patients to be included was supported by sample calculation; however, subgroup analysis should be interpreted carefully considering the heterogeneity of the population.

Although Ag-RDTs presented lower sensitivity than RT–qPCR, those tests may be a useful diagnostic tool for COVID-19, rapidly detecting patients with high viral loads. These results confirm that the performance of rapid tests based on the antigen search for SARS-COV-2 in the routine of laboratories or health services may be inferior to that described by the manufacturers and that marked differences exist between commercial brands. Viral load seems to be the main determinant of test positivity, explaining the influence of symptom duration on observed performance. Even so, a positive Ag-RDT remains useful to diagnose symptomatic cases at hospital admission, particularly in terms of the speed of results, considering that a negative result does not rule out SARS-CoV-2 infection. The main benefit of Ag-RDTs would be to confirm the COVID-19 diagnosis in patients with higher viral shedding and possibly greater infectivity, reducing the number of cases for RT–qPCR. Thus, diagnostic algorithms combining tests with different methodologies must be evaluated in cost-effectiveness studies to confirm the best strategy for using rapid tests. Therefore, the various tests must be performed in different populations, justifying further studies in real-life scenarios, such as this one.

## Supporting information

S1 Fig STARD flow diagram showing the flow of patients included in the study and it primary outcome. (TIF)

S1 Table Complementary analysis of patients simultaneously undergoing the test from the COPAN^®^ medium and directly from the swab. (DOCX)
