# Supplementary material for: Performance differences among commercially available antigen rapid tests for COVID-19 in Brazil
Source: PLoS One. 2022 Jun 16;17(6):e0269997. doi: 10.1371/journal.pone.0269997 (PMC9202877; doi:10.1371/journal.pone.0269997)
Supplement: S1 Table — (DOCX) [file pone.0269997.s002.docx]

| **Testes** | | | **Swab directly in buffer** | | **Agreement** | ***Kappa*** |
| --- | --- | --- | --- | --- | --- | --- |
|  |  |  | **Positive** | **Negative** |  |  |
| **COVI-19 Ag ECO Teste (ECO Diagnostica)** | **UTM** | **Positive** | 2 | 3 | 62.5% | 0.127 |
|  |  | **Negative** | 3 | 8 |  |  |
| **SARS-CoV-2 Ag-RDT (SD Biosensor)** | **UTM** | **Positive** | 6 | 0 | 62,5% | 0.333 |
|  |  | **Negative** | 6 | 4 |  |  |
| **CORIS Bioconcept® COVID-19 Ag-RDT (Nanosens)** | **UTM** | **Positive** | 1 | 1 | 75% | 0.250 |
|  |  | **Negative** | 2 | 8 |  |  |

**S1 Table.** Complementary analysis of patients simultaneously undergoing the test from the COPAN® medium and directly from the swab
